# Chd1 co-localizes with early transcription elongation factors independently of H3K36 methylation and releases stalled RNA polymerase II at introns

**DOI:** 10.1186/1756-8935-7-32

**Published:** 2014-10-27

**Authors:** Daechan Park, Haridha Shivram, Vishwanath R Iyer

**Affiliations:** Center for Systems and Synthetic Biology, Institute for Cellular and Molecular Biology, Department of Molecular Biosciences, University of Texas, 2500 Speedway, Austin, TX 78712 USA

**Keywords:** Chromodomain helicase DNA binding protein 1 (Chd1), Chromatin remodeling, Transcription elongation, H3K36 methylation, Intron

## Abstract

**Background:**

Chromatin consists of ordered nucleosomal arrays that are controlled by highly conserved adenosine triphosphate (ATP)-dependent chromatin remodeling complexes. One such remodeler, chromodomain helicase DNA binding protein 1 (Chd1), is believed to play an integral role in nucleosomal organization, as the loss of Chd1 is known to disrupt chromatin. However, the specificity and basis for the functional and physical localization of Chd1 on chromatin remains largely unknown.

**Results:**

Using genome-wide approaches, we found that the loss of Chd1 significantly disrupted nucleosome arrays within the gene bodies of highly transcribed genes. We also found that Chd1 is physically recruited to gene bodies, and that its occupancy specifically corresponds to that of the early elongating form of RNA polymerase, RNAPII Ser 5-P. Conversely, RNAPII Ser 5-P occupancy was affected by the loss of Chd1, suggesting that Chd1 is associated with early transcription elongation. Surprisingly, the occupancy of RNAPII Ser 5-P was affected by the loss of Chd1 specifically at intron-containing genes. Nucleosome turnover was also affected at these sites in the absence of Chd1. We also found that deletion of the histone methyltransferase for H3K36 (*SET2*) did not affect either Chd1 occupancy or nucleosome organization genome-wide.

**Conclusions:**

Chd1 is specifically recruited onto the gene bodies of highly transcribed genes in an elongation-dependent but H3K36me3-independent manner. Chd1 co-localizes with the early elongating form of RNA polymerase, and affects the occupancy of RNAPII only at genes containing introns, suggesting a role in relieving splicing-related pausing of RNAPII.

**Electronic supplementary material:**

The online version of this article (doi:10.1186/1756-8935-7-32) contains supplementary material, which is available to authorized users.

## Background

Nucleosomes represent the basic unit of chromatin and their post-translational modifications and positions play a critical role in both the structure and transcriptional regulation of chromatin [1]. High resolution genome-wide maps of nucleosome positions reveal two key features: (i) a nucleosome depleted region (NDR) flanked by -1 and +1 nucleosomes and (ii) well-positioned nucleosomes separated at regular distances by linker DNA [2–4]. *In vitro* DNA-histone reconstitution assays and *in vivo* micrococcal nuclease (MNase) digestion experiments have shown that preferred DNA sequences and structural features on nucleosomes determine nucleosomal organization [3, 5, 6]. In addition, ATP-dependent chromatin remodeling complexes are key determinants of nucleosome organization [7]. High-resolution mapping of chromatin remodelers shows a high degree of specificity relative to nucleosomes [8].

Overall nucleosome positions across the genome are typically not strongly disrupted by the loss of a single chromatin remodeler yet tend to be significantly disrupted by double or triple deletions [9–11], suggesting that chromatin remodeling complexes operate with redundant functionality. Exceptions to this trend, however, can be observed. For example, in contrast to other chromatin remodelers, the singular loss of Chd1 severely disrupts well-organized nucleosome arrays in yeast [10, 12, 13]. However, a recent high-resolution study of the occupancy profiles of various chromatin remodelers in yeast did not include Chd1, so the basis for its role in nucleosome organization is still largely unknown [8].

In *Schizosaccharomyces pombe*, nucleosome arrays at highly transcribed genes become disorganized in a strain deleted for the ortholog of Chd1 (i.e. *hrp3Δ*) [13]. A more recent high-throughput study also in *S. pombe,* however, showed that genes with high and low transcription rates were equally disrupted [12]. Though conflicting in their interpretations, the two papers actually reported very similar nucleosome profiles. This discrepancy in part reflects the lack of a definitive quantitative method for the comparison of genome-wide MNase-seq datasets of nucleosome positions.

Studies on individual genes have shown that Chd1 localizes on highly transcribed genes and interacts with transcription elongation factors [14, 15]. Consistent with these observations, Chd1 ChIP-seq confirmed the localization of Chd1 within gene bodies and with high enrichment at highly transcribed genes [10]. Interestingly, the average nucleosome profile of *chd1Δ* showed that the extent of disruption was particularly strong at the +2 and more downstream nucleosomes, implying that Chd1 works in nonpromoter regions [10, 12, 13]. However, a recent report showed that Chd1 also binds to promoters in addition to gene bodies [16]. Resolving such conflicting data on Chd1 occupancy remains challenging because the molecular mechanism by which Chd1 is recruited to chromatin remains unknown.

Chd1 has a double chromodomain motif, which in other proteins typically mediates interactions with methylated peptides such as tri-methylated histone H3K4 (i.e. H3K4me3) [17]. However, detailed analysis of yeast Chd1 suggested that it is incapable of interacting directly with H3K4me3 [18, 19]. Some classes of chromodomain proteins can bind to H3K36me3 in conjunction with PHD finger domains [20], but *in vitro*, Chd1 binds to nucleosomes methylated and unmethylated at H3K36 with a similar affinity [21]. H3K4me3 is a mark of promoters and early transcription elongation whereas H3K36me3 marks gene bodies [22], which is where Chd1 localization has been observed. Recent mass spectrometry analyses of H3K36me3 immunoprecipitates from mononucleosomes have linked Chd1 to H3K36me3 [23]. Deletion of *CHD1* has been shown to cause a shift in the distribution of H3K36me3 upstream in gene bodies [23, 24], suggesting that Chd1 plays a role in maintaining the positioning of H3K36me3. These studies taken together suggest that Chd1 localization within gene bodies could be mediated either directly or indirectly by H3K36me3.

In this study, we first quantitatively compared nucleosome occupancy profiles between wild-type (WT) and *chd1Δ* strains. Our novel approach revealed that the deletion of *CHD1* specifically disrupts nucleosomal organization at highly transcribed genes. Chd1 occupancy at highly transcribed genes is strikingly similar to phosphorylated RNAPII at Ser 5. Interestingly, RNAPII Ser 5-P occupancy was dramatically altered but specifically at intron-containing genes in the absence of Chd1. Finally, we tested the possibility that Chd1 recruitment is mediated by H3K36me3 by examining Chd1 occupancy in a strain lacking the histone methyltransferase SET2, and found that its recruitment to transcribed regions occurs independently of H3K36me3.

## Results and discussion

### Nucleosome organization is severely disrupted at highly transcribed genes in *chd1Δ*mutants

**Figure 1:**
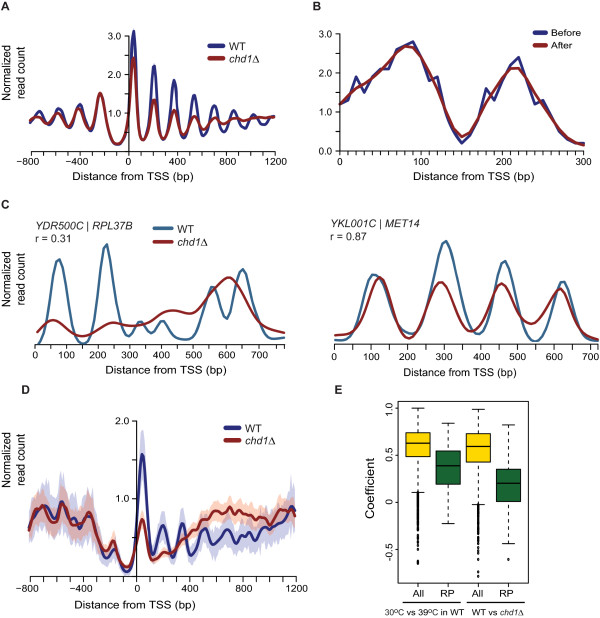
**Nucleosome occupancy in** ***chd1Δ.*** **(A)** Average nucleosome profile for all genes (n =5,207) shows that nucleosome occupancy is reduced at gene bodies in *chd1Δ*. The X-axis shows genomic position relative to the transcription start site (TSS). The Y-axis represents average read counts per million reads (M). **(B)** Example of smoothing MNase-seq read data using a spline function. Smoothing removes the noise caused by fluctuations in read coverage that can give erroneous correlation coefficients. Blue line is the raw, binned read count data and the red line is smoothed **(C)** Smoothing followed by Pearson correlation, called shapeDiff analysis, measures the nucleosome occupancy similarity as 0.31 between wild-type (WT) and *chd1Δ* over the transcribed region of *RPL37B. MET14* has a high correlation coefficient between WT and *chd1Δ* as determined by shapeDiff due to high similarity of nucleosome occupancy profiles. **(D)** Average nucleosome profile for RP genes (n =136) shows strong nucleosome disruption upon deletion of *CHD1*. The shaded bands represent the 95% confidence interval of the data. **(E)** Quantitation of nucleosome disruption at ribosomal protein (RP) genes after heat shock or upon deletion of *CHD1* (*chd1Δ*). The Y axis shows the correlation coefficient as measured by shapeDiff for genes in each class as indicated on the X-axis.

We used MNase-seq to map nucleosome positions in wild-type (WT) and *chd1Δ* strains of budding yeast [4]. We found that the loss of Chd1 disrupted nucleosome organization within gene bodies, consistent with previous observations in both budding and fission yeast [10, 12, 13] (Figure 1A). We further confirmed this phenotype in the *chd1Δ* strain with a different resistance marker (Methods, [see Additional file 1: Figure S1A]). Although these genome-wide profiles indicate that nucleosome occupancy is generally affected by Chd1, they do not reveal which subsets of genes are specifically dependent on Chd1 function, and what the molecular basis of this dependency might be. To gain insights into these questions, we developed an approach based on quantitatively scoring all genes by the extent of nucleosome disruption.In this approach, which we called ‘shapeDiff analysis,’ we first smoothed nucleosome occupancy signals based on read counts using a spline function, then measured the correlation coefficient between the WT and mutant nucleosome profiles for every gene in the genome (Methods). This approach has the advantage that the correlation measurement is relatively insensitive to noisy fluctuations in the nucleosome occupancy signal caused by low read counts (Figure 1B).

In our first experiments using shapeDiff analysis, we began by comparing nucleosome profiles of WT and *chd1Δ* strains, focusing on the region between the transcription start site (TSS) and the polyadenylation site (PAS) [25]. This analysis revealed that shapeDiff could efficiently identify genes with similar or disrupted nucleosome occupancy patterns. For example, *MET14* has four distinct nucleosomes within the gene body, which could be detected in both the WT and in *chd1Δ*; in accordance with the small nucleosome shifts observed between the two strains, shapeDiff measured a high correlation for the nucleosomes (Figure 1C). In contrast, the occupancy of the +1 and +2 nucleosomes for *RPL37B* were visibly dramatically reduced in *chd1Δ*, and shapeDiff showed that nucleosomal periodicity disappeared altogether at the 3′ end of the gene, resulting in a much lower correlation coefficient (Figure 1C).

Genome-wide, the correlation of nucleosome profiles between WT and *chd1Δ* reported by shapeDiff was significantly lower at highly transcribed genes, indicating that the loss of Chd1 leads to greater nucleosome disorganization at loci with high transcription rates (see Additional file 1: Figure S1B). In order to understand the relative magnitude of the disruption caused by *chd1Δ*, we compared it to heat shock - a perturbation also known to disrupt the nucleosome organization of highly transcribed genes [4]. Specifically, we analyzed the correlation among ribosomal protein (RP) genes under normal conditions and heat shock as well as the correlation among RP genes in WT and *chd1Δ* strains. RP genes serve as a good comparative measure for nucleosome occupancy affected by *CHD1* deletion because they exhibit high transcription rates (TR), are strongly repressed by heat shock, and show significant nucleosome depletion [4, 26]. RP genes showed a higher correlation of nucleosome occupancies between normal and heat shock conditions than between WT and *chd1Δ* (Figure 1D and E). These data suggest that the deletion of *CHD1* more strongly depleted nucleosome arrays at highly transcribed genes than acute heat shock did, and raise the question of how Chd1 occupancy relates to its observed effects on a subset of genes.

### The Chd1 binding profile is similar to the early elongating form of RNAPII phosphorylated at Ser-5

**Figure 2:**
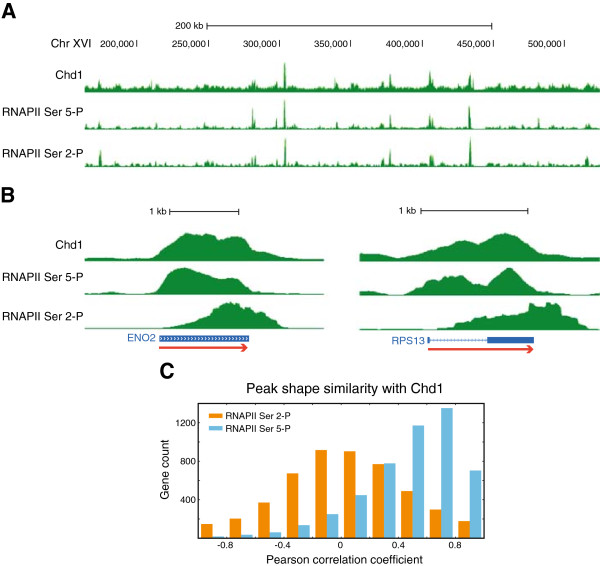
**Chd1 co-localizes with early elongating RNAPII. (A)** In a wide view, Chd1 occupancy appears similar to both RNAPII Ser 5-P and Ser 2-P occupancy. **(B)** Close-up views of highly expressed genes reveal that Chd1 occupancy appears similar to the occupancy profile of RNAPII Ser 5-P, but not Ser 2-P. **(C)** The peak shapes of Chd1 are quantitatively compared with those of either RNAPII Ser 5-P or Ser 2-P using shapeDiff. Histogram of correlation coefficients shows that RNAPII Ser 5-P has high correlation with Chd1 occupancy, whereas RNAPII Ser 2-P has little correlation with Chd1 on a genome wide scale.

Recent studies examining Chd1 occupancy using low-throughput and genomic approaches give conflicting data regarding its occupancy at promoters, gene bodies or both regions of genes [10, 13, 14, 16]. We performed ChIP-seq using a Myc-tagged Chd1 strain to determine its occupancy. Our data were consistent with the study of Gkikopoulos T *et al*. (see Additional file 1: Figure S2) [10]. Given the effect of Chd1 on nucleosome occupancy at highly transcribed genes that we had observed earlier, we wanted to assess the relationship of Chd1 binding to markers of transcriptional activity. We, therefore, compared Chd1 binding to that of different forms of elongating RNA polymerase that we measured separately using ChIP-seq. At the chromosomal scale, Chd1 occupancy appeared similar to both RNAPII Ser 5-P and RNAPII Ser 2-P occupancy (Figure 2A). Moreover, Myc-tagged Chd1 could co-immunoprecipitate both RNAPII Ser 5-P and Ser 2-P from cell extracts, suggesting an *in vivo* association (see Additional file 1: Figure S3). However at individual genes, the Chd1 binding pattern was strikingly similar to the pattern of RNAPII Ser 5-P occupancy but not to that of RNAPII Ser 2-P (Figure 2B). To quantify this similarity in binding patterns, we carried out shapeDiff analysis of Chd1 and RNAPII occupancy over genes. Chd1 binding peak shapes were more strongly correlated with RNAPII Ser 5-P (median =0.54) than with RNAPII Ser 2-P (median =0.04) (Figure 2C). The 2,056 genes had a correlation coefficient greater than 0.6 in the peak shape comparison between Chd1 and RNAPII Ser 5-P. The distribution of the peak shape correlations confirmed that Chd1 is co-localized with an early transcription elongation factor (RNAPII Ser 5-P) rather than a late transcription elongation factor (RNAPII Ser 2-P).

### The loss of Chd1 specifically affects RNAPII Ser 5-P primarily at intron-containing genes

**Figure 3:**
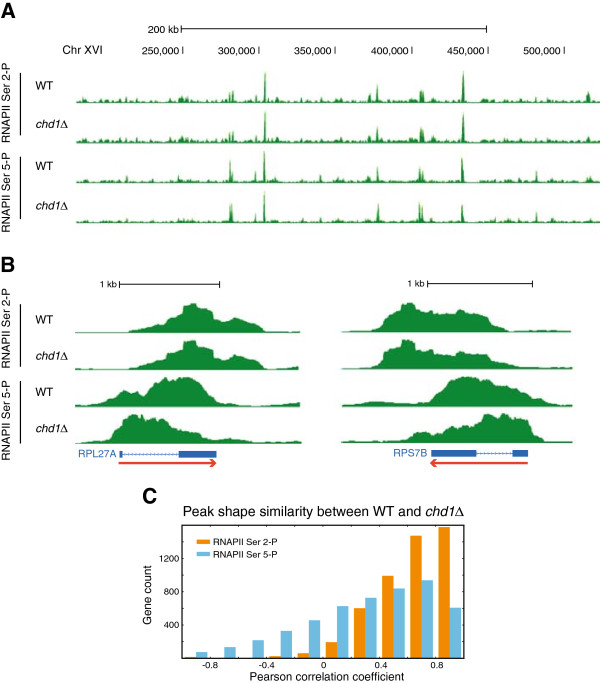
**Loss of Chd1 leads to changes in local occupancy of RNAPII Ser 5-P. (A)** Wide view shows no large-scale occupancy changes of either form of elongating RNAPII between wild-type (WT) and *chd1Δ*. **(B)** Close-up of highly transcribed genes showing that RNAPII Ser 5-P shifts upstream in *chd1Δ*, but RNAPII Ser 2-P peaks appear very similar between WT and *chd1Δ*. **(C)** shapeDiff quantification of the peak shape similarity of RNAPII Ser 5-P and RNAPII Ser 2-P between WT and *chd1Δ*. The effect of *CHD1* deletion on changes in peak shape is much stronger for RNAPII Ser 5-P than for RNAPII Ser-2 P.

It is known that overall gene expression levels are only slightly altered by the loss of Chd1 [27, 28], but cryptic and antisense transcription is notably increased [12, 13, 23, 29, 30]. Based on these observations and our results above, we hypothesized that while the loss of Chd1 does not lead to partial or complete loss of elongating RNAPII, its local positioning is affected. To test this hypothesis, we measured the occupancies of RNAPII Ser 5-P and RNAPII Ser 2-P separately in a *chd1Δ* strain. In the broad genomic view, we did not observe dramatic changes in the occupancy of either form of elongating RNAPII (Figure 3A). When we examined individual genes, however, the binding peak shapes of RNAPII Ser 5-P were disrupted at some highly transcribed genes, especially RP genes (Figure 3B). In order to quantitate the peak shape similarities of elongating RNAPII between WT and *chd1Δ* strains on a genome-wide scale, we again used shapeDiff to generate correlations for each gene. Interestingly, RNAPII Ser 2-P was relatively unaffected by the loss of Chd1 (median correlation =0.69), but RNAPII Ser 5-P peak shapes were clearly affected by the deletion of *CHD1* (median correlation =0.38) (Figure 3C).

**Figure 4:**
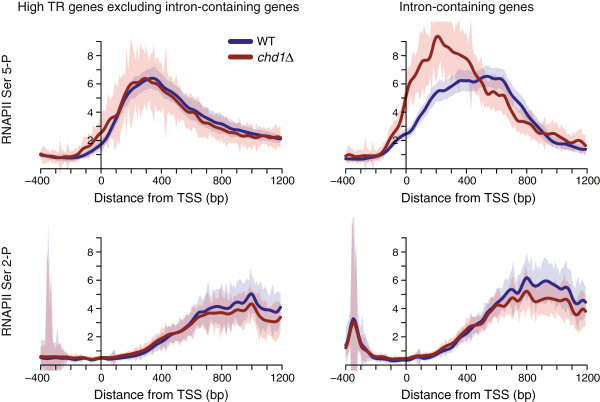
**Intron-containing genes show a marked shift in the occupancy of RNAPII Ser 5-P in the absence of Chd1.** The top 500 genes with the highest transcription rate (TR) as measured by RNAPII Ser-5 P occupancy [35] were defined as the high TR genes. Upper and lower panels show average profiles of RNAPII Ser 5-P and Ser 2-P occupancies, respectively. The left panels show the high TR gene set without any intron-containing genes (n =382), whereas the right panels show only the intron-containing genes from among the high TR genes (n =118). The shaded bands represent the 95% confidence interval of the data.

**Figure 5:**
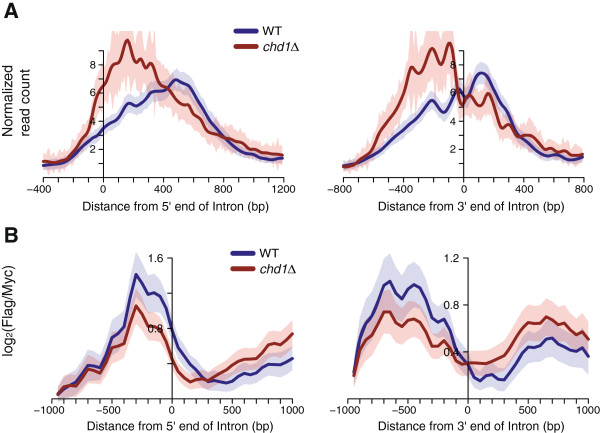
**The stalling of RNAPII Ser 5-P and decreased histone exchange in the absence of Chd1 is evident at the 3′ end of introns. (A)** Average profile of RNAPII Ser 5-P for intron-containing genes among the high TR genes (n =118), aligned by the 5′ end and 3′ end of introns. **(B)** Histone exchange rate in WT and *chd1Δ* strains from Smolle *et al*. was aligned by the 5′ end and 3′ end of introns [23].

Since we had observed strong Chd1 binding on the genes with high transcription rate (TR), we examined the effect of *CHD1* deletion on the peak shapes of RNAPII Ser 5-P at the subset of high TR genes. Interestingly, the average peak shapes of RNAPII Ser 5-P were shifted toward the 5′ end of the high TR genes in *chd1Δ* relative to WT cells (see Additional file 1: Figure S4A). In *S. cerevisiae*, high TR genes include a considerable number of intron-containing genes. In order to identify the primary determinant of Chd1 sensitivity, we separated the high TR intron-less and intron-containing genes. Strikingly, intron-containing genes showed substantial changes in RNAPII Ser 5-P occupancy, whereas the high TR genes excluding intron-containing genes had little alteration in the *chd1Δ* mutant (Figure 4). When the RNAPII Ser 5-P occupancy for the high TR intron-containing genes was aligned by exon-intron junctions, the shift to the upstream direction in *chd1Δ* was more evident at 3′ end of introns (Figure 5A). Recent studies have shown that introns result in pausing of RNAPII [31–33], and independently, that Chd1 relieves RNAPII stalling at promoters in mammalian cells [34]. Our data suggest that Chd1 could additionally relieve the stalling of the early elongating form of RNAPII at introns.

To explore a possible mechanistic link between RNAPII Ser 5-P stalling and loss of Chd1, we analyzed previously reported histone exchange data in WT and *chd1Δ* strains [23]. Smolle *et al*. measured the histone exchange rate by using yeast strains that expressed constitutive Myc-tagged H3 and galactose-inducible Flag-tagged H3. The histone exchange rate was defined as the occupancy levels of Flag-H3 relative to Myc-H3. As reported by Smolle *et al*., we confirmed that nucleosome exchange is slower at 5′ end but faster at the 3′ end of genes in *chd1Δ* (see Additional file 1: Figure S4B) [23, 24]. Interestingly, we found that loss of Chd1 reduces nucleosome turnover at the 3′ end of introns. This exchange rate was restored at the 3′ intron-exon junction and even became higher downstream of the 3′ intron-exon junction in *chd1Δ* (Figure 5B), suggesting that a role of Chd1 in facilitating nucleosome exchange at the 3′ end of introns allows the processivity of RNAPII Ser 5-P at the junctions.

### Recruitment and function of Chd1 at gene bodies is independent of H3K36 methylation

Our data raise the question of how Chd1 is recruited to gene bodies, where we found that it increases steady state nucleosome occupancy and affects RNAPII. There are several reasons that its interaction with gene bodies could be dependent on H3K36me3. First, Chd1 was shown to be associated with H3K36me3 by mass spectrometry [23], though this interaction is likely to be indirect. Second, the H3K36me3 signal was shifted upstream by the loss of Chd1 whereas H3K4me3 showed no change [23, 24]. Third, we found that nucleosome disruption in *chd1Δ* occurred mainly at the +2 and subsequent downstream nucleosomes, where H3K36 methylation is more abundant than H3K4 methylation [10]. Fourth, H3K36me3 is associated with the gene bodies of highly transcribed genes during transcription elongation, which coincides with the region where we observed Chd1 localization to [36–38]. Fifth, RNAPII Ser 5-P, which we showed is specifically related to Chd1 occupancy, has been co-purified with the H3K36 methyltransferase Set2 [39].

**Figure 6:**
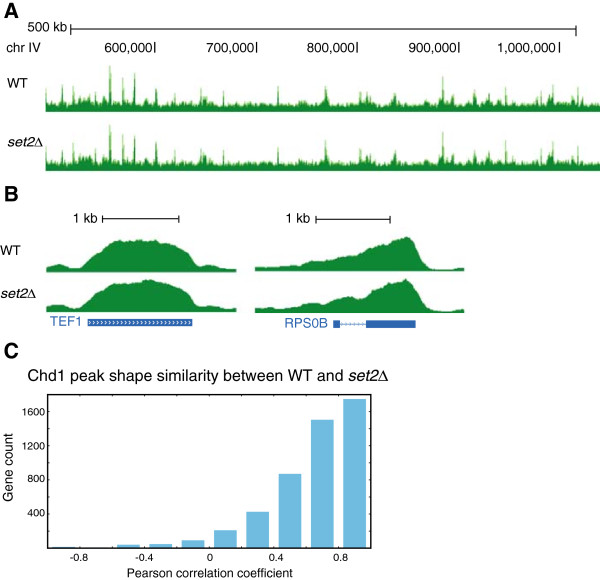
**Chd1 localization on chromatin is Set2-independent. (A, B)** The binding profiles of Chd1 are indistinguishable between WT and *set2Δ*. **(C)** shapeDiff analysis confirms that *set2Δ* has little effect on Chd1 occupancy on a genome wide scale.

Based on these lines of evidence, we hypothesized that the critical function of Chd1 at gene bodies is mediated by its recruitment in an H3K36me3-dependent manner. Although Chd1 occupancy has been reported to be independent of H3K36 methylation, no published data is available, and it is unclear how the genome-wide occupancy of Chd1 is affected by levels of H3K36 methylation [30]. To test our hypothesis, we measured the genome-wide occupancy of our epitope-tagged Chd1 after we deleted the H3K36 methyltransferase gene *SET2*. Examination of the ChIP-seq data confirmed the successful deletion of *SET2* in that only a few reads were mapped to the *SET2* gene body (see Additional file 1: Figure S5A). Immunoblotting revealed that the levels of H3K36me3 were greatly reduced by loss of Set2, while Chd1 expression levels were unaltered (see Additional file 1: Figure S5B). However, global Chd1 occupancy in *set2Δ* appeared identical to that in WT (Figure 6A). No significant changes were detectable in the pattern of Chd1 occupancy at several individual genes (Figure 6B). Additionally, shapeDiff analysis also revealed a high similarity between Chd1 occupancy in WT and *set2Δ* (median correlation coefficient =0.71) (Figure 6C), which was comparable to the similarity of two independent ChIP-seq datasets that also measured Chd1 occupancy in a WT strain (median correlation coefficient =0.65). Thus, loss of Set2 had no effect on Chd1 occupancy, suggesting that Chd1 occupancy within gene bodies is Set2-independent. This observation is also supported by the fact that *set2Δ* shows normal nucleosome organization and does not recapitulate the loss of Chd1 [see Additional file 1: Figure S6A and S6B]. Therefore, we conclude that Chd1 is recruited onto chromatin in a H3K36 methylation-independent manner, and that methylation at H3K36 has no effect on well-organized nucleosome arrays. Based on the known interacting partners of Chd1 such as the PAF and SAGA complexes and Spt5 [14, 15, 17], it is instead possible that early transcription elongation factors are likely to recruit Chd1 to gene bodies.

## Conclusions

We developed a quantitative method, shapeDiff, to compare nucleosome and DNA binding protein occupancies on chromatin. By applying shapeDiff to genome-wide occupancy and chromatin data, we clarified the physical and functional specificity of the chromatin remodeler Chd1 [10, 12, 13, 16]. We showed that the role of Chd1 in the organization of nucleosome arrays is critical specifically within the gene bodies of highly transcribed genes. We also showed that Chd1 physically co-localizes with the early form of elongating RNA polymerase (RNAPII Ser 5-P), but not with its late elongating form. Moreover, the early elongating RNAPII Ser 5-P was specifically shifted upstream by the loss of Chd1. Remarkably, the upstream shift of RNAPII Ser 5-P was seen most strongly at exon-intron junctions, suggesting that Chd1 normally functions to relieve stalled RNAPII at introns. Finally, we showed that Chd1 occupancy is independent of the methylation levels of histone H3K36, suggesting that its recruitment to chromatin is not mediated by an interaction of its chromodomain with the landmark histone modification of transcribed regions.

## Methods

### Yeast strains and cell culture

The *S. cerevisiae* strain BY4741 (MATa *his3Δ1 leu2Δ0 met15Δ0 ura3Δ0*) was used as a wild type strain and background genotype for *chd1Δ* and *set2Δ.* The *chd1Δ* strain in Additional file 1: Figure S1A was obtained from the yeast deletion collection (Open Biosystems, now GE Dharmacon, Lafayette, CO, USA) [40]. For *chd1Δ* strain in Figure 1A, we deleted *CHD1* by replacing the protein coding region of *CHD1* with the His3MX6 cassette.

All cells were cultured in YPD (yeast extract, peptone, dextrose) media at 30°C to an A600 OD of 0.8 with shaking at 250 rpm. For heat shock, the mid-log cells were harvested, re-suspended in pre-warmed YPD media, incubated in 39°C water bath for 15 mins, treated with formaldehyde, and then stored at -80°C. To tag endogenous Chd1, a 13Myc-His3MX6 cassette was amplified from pFA6a-13Myc-His3MX6, and transformed into WT or *set2Δ* [41]. The cassettes were integrated into the *CHD1* stop codon, and 13XMyc at the C terminal of Chd1 was confirmed by PCR and immunoblot.

### Immunoblotting

Whole cell extracts were prepared from 30 ml cultures of 0.8 OD WT and *set2Δ* cells carrying endogenous 13XMyc tagged Chd1. Then, 30 μl of each extract was run on a 4 to 20% gradient SDS-polyacrylamide gel and transferred to a polyvinyl difluoride (PVDF) membrane. In order to confirm 13XMyc tagging of Chd1 and compare the levels of Chd1 expression between WT and *set2Δ,* we detected Chd1 using horseradish peroxidase (HRP)-conjugated c-Myc antibody (Santa Cruz Biotechnology, Dallas, TX, USA, 9E10, cat.# sc-40). Levels of H3K36me3 in *set2Δ* were examined by probing with anti-Histone H3 (tri methyl K36) antibody (Abcam, Cambridge, MA, USA, cat# ab9050), and GAPDH antibody (Santa Cruz Biotechnology, Dallas, TX, USA, FL-335, cat.#sc-25778) was used to visualize the loading control proteins.

### Chromatin immunoprecipitation

All 150-ml cell cultures were treated with formaldehyde to be a final concentration 1% for 15 min, then quenched with glycine to a final concentration of 125 mM for 5 min. The DNA-protein complexes were sheared by ultra-sound sonication, then incubated overnight with 100 μl of anti-Myc conjugated agarose beads (Sigma Aldrich, St. Louis, MO, USA, cat.# E6654), 8 μg of RNAPII Ser 5-P specific antibody (Abcam, Cambridge, MA, USA, cat.# ab5131), and 8 μg of RNAPII Ser 2-P specific antibody (Abcam, Cambridge, MA, USA, cat.# ab5095) to pull down Chd1, RNAPII Ser 5-P and, Ser 2-P, respectively. Then, for the RNAPII ChIP, 100 μl of pre-washed protein A beads were added and incubated for 4 hours. After serial wash steps, immunoprecipitated DNA was recovered with overnight incubation at 65°C water bath followed by ethanol precipitation. Subsequently, sequencing libraries were prepared using NEBNext ChIP-Seq Library Prep Master Mix Set (New England Biolabs, Ipswich, MA, USA, cat.# E6240L) and Bioo multiplex adapter for Illumina (Bioo Inc, Austin, TX USA), then sequenced on Illumina HiSeq 2000.

### Mononucleosome isolation

We followed the mononucleosome isolation protocol described in [4]. Briefly, cells were prepared as described above for ChIP by the quenching step and resuspended in 20 ml of zymolyase buffer. Next, 250 μg of zymolyase (MP Biomedicals, Santa Ana, CA, USA, cat.# IC320921) was added to make spheroplasts, then resuspended in 2 ml NP buffer. The spheroplasts were treated with MNase (Worthington Biochemical Corp., Lakewood, NJ, USA, cat.# LS004797) at a concentration from 40 U-100 U for 10 min at 37°C. The DNA-protein complexes were reverse-crosslinked in 10 mM EDTA and 1% SDS buffer with Proteinase K at 65°C overnight. RNA was removed by RNase A treatment, then DNA was extracted with phenol-chloroform and purified by ethanol precipitation. Finally, DNA was run on an E-gel system (Invitrogen, Carlsbad, CA, USA), and approximately 147-bp DNA fragments were size-selected. Library preparation and sequencing were performed as described above for ChIP-seq.

### Bioinformatics and shapeDiff analysis

Sequencing reads were mapped onto the sacCer3 reference genome using the Burrows-Wheeler Aligner (BWA, version 0.6.2) with default options [42]. Wig files were generated from the bam files and loaded on our UCSC Genome Browser mirror for visual analysis. To quantitatively analyze differences in nucleosome organization between WT and *chd1*Δ strains, we developed a novel analysis pipeline. MNase-seq has been widely used for mapping nucleosome occupancy, but quantifying differences in organization is challenging for several reasons. First, the variable strength of nucleosomal signals in MNase-seq complicate any quantitative comparisons; standard peak calling methods that are more suited for ChIP-seq data rely upon the ability to measure high, individual, and dispersed peaks. Second, the total number of nucleosomes detected is correlated with sequencing depth [43], thus differences arising from sequence coverage can be misinterpreted as nucleosome depletion or acquisition when two MNase-seq data sets are compared. Third, peak height can vary due to artifacts that occur as the result of different MNase digest concentrations [44]. However, most of these artifacts have little effect on nucleosomal periodicity.

The Pearson correlation has been used to quantitatively compare MNase-seq profiles for levels of nucleosome occupancies at a given locus [44, 45]. By focusing on overall comparative trends rather than signal strength at a single site, the Pearson correlation mitigates the effect of many MNase artifacts. However, noisy signals due to low sequencing coverage at certain genes could result in erroneously low correlations. In order to overcome this problem, we smoothed nucleosome occupancy signals using a spline function as a preliminary step before the Pearson correlation (Figure 1B). An advantage of this approach is that, due to the nature of the Pearson correlation, the process of normalizing sequencing depth (that is, multiplying or dividing read count signal by a scaling factor) should not affect any correlation calculations.

For shapeDiff analysis, genomic regions between transcription start sites (TSS) and polyadenylation sites (PAS) were divided into bins of 10 bp, and reads were counted. Then, the counts were smoothed using the built-in spline function in R with default parameters (R version 3.0.2). For a given gene, the Pearson correlation coefficient was calculated for the smoothed counts between two samples. This process was iterated for every gene for which TSS and PAS coordinates were available [25].

#### Accession codes

The ChIP-seq data from this study have been deposited in the Gene Expression Omnibus (GEO) database under accession number GSE56061. The MNase-seq data are also available from GEO as accession number GSE56095.

## Electronic supplementary material

Additional file 1: **A PDF document containing six supplementary figures.** (PDF 519 KB)

## Abbreviations

ChIP: chromatin immunoprecipitation
H3K4me3: tri-methylated histone H3 lysine 4
H3K36me3: tri-methylated histone H3 lysine 36
HRP: horseradish peroxidase
MNase: micrococcal nuclease
PAS: polyadenylation site
PVDF: polyvinyl difluoride
RNAPII: RNA polymerase II
RP: ribosomal protein
SAGA: Spt-Ada-Gcn5 acetyltransferase
Ser 2-P: phosphorylated at Ser-2
Ser 5-P: phosphorylated at Ser-5
TR: transcription rates
TSS: transcription start site
TSS: transcription start site
WT: wild-type.
