# Supplementary material for: Chd1 co-localizes with early transcription elongation factors independently of H3K36 methylation and releases stalled RNA polymerase II at introns
Source: Epigenetics Chromatin. 2014 Oct 27;7:32. doi: 10.1186/1756-8935-7-32 (PMC4230344; doi:10.1186/1756-8935-7-32)
Supplement: Supplementary file 1 — Additional file 1: A PDF document containing six supplementary figures. (PDF 519 KB) [file 13072_2014_334_MOESM1_ESM.pdf]

Supplemental figures

Figure S1 (A) Nucleosome disruption is independently observed in a strain deleted for *CHD1* by an alternative G418 resistance marker. (B) Box plot of correlation coefficients measured by shapeDiff showing nucleosome arrays are significantly disrupted at the gene bodies of highly transcribed genes in *chd1Δ*.

Figure S2 Our Chd1 occupancy data (bottom track in each panel) is consistent with published data from the study of Gkikopoulos T et al. (*Science* 2011, 333:1758-1760) (A) Wide view, (B) Close-up view of two example genes.

Figure S3 Myc-Chd1 was immunoprecipitated with anti-Myc conjugated agarose beads, then the purified protein complexes were probed with antibodies detecting RNAPII Ser 5-P and Ser 2-P, respectively.

Figure S4 (A) Average profiles of RNAPII Ser 5-P and Ser 2-P occupancy for the high TR genes. The shaded bands represent the 95% confidence interval of the data. The average peak shapes of RNAPII Ser 5-P show a small shift toward 5' end in *chd1Δ* relative to WT cells. (B) Average profiles of histone exchange rate for all genes in WT and *chd1Δ*, aligned by the 5' end and 3' end of introns. Histone exchange data were obtained from Smolle T et al (*Nat Struct Mol Biol* 2012, **19:**884-892).

Figure S5 (A) Chd1 ChIP-seq in WT and *set2Δ*. Lack of sequencing reads on the *SET2* genomic region confirms successful deletion of *SET2* in the strain used to generate Chd1 ChIP-seq data. (B) Deletion of *SET2* does not affect the expression levels of 13X-Myc-tagged Chd1, but significantly reduces H3K36me3 levels.

Figure S6 (A) Average nucleosome profile shows high similarity between replicate WT samples. The similarity between WT and *set2Δ* is comparable to independent replicate WT samples. (B) Peak shape comparison by shapeDiff

shows that nucleosome occupancy in *set2Δ* is quantitatively indistinguishable from replicates.

Figure S1

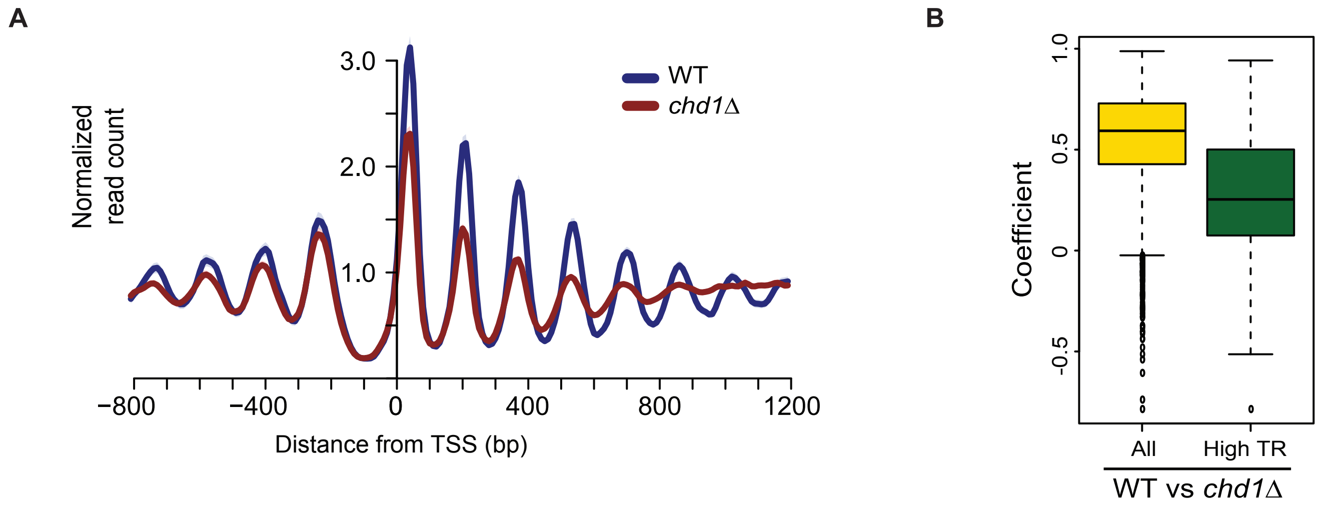
A
B
WT
chd1Δ
Normalized read count
3.0
2.0
1.0
−800
−400
0
400
800
1200
Distance from TSS (bp)
Coefficient
1.0
0.5
0
-0.5
All
High TR
WT vs chd1Δ

Figure S2

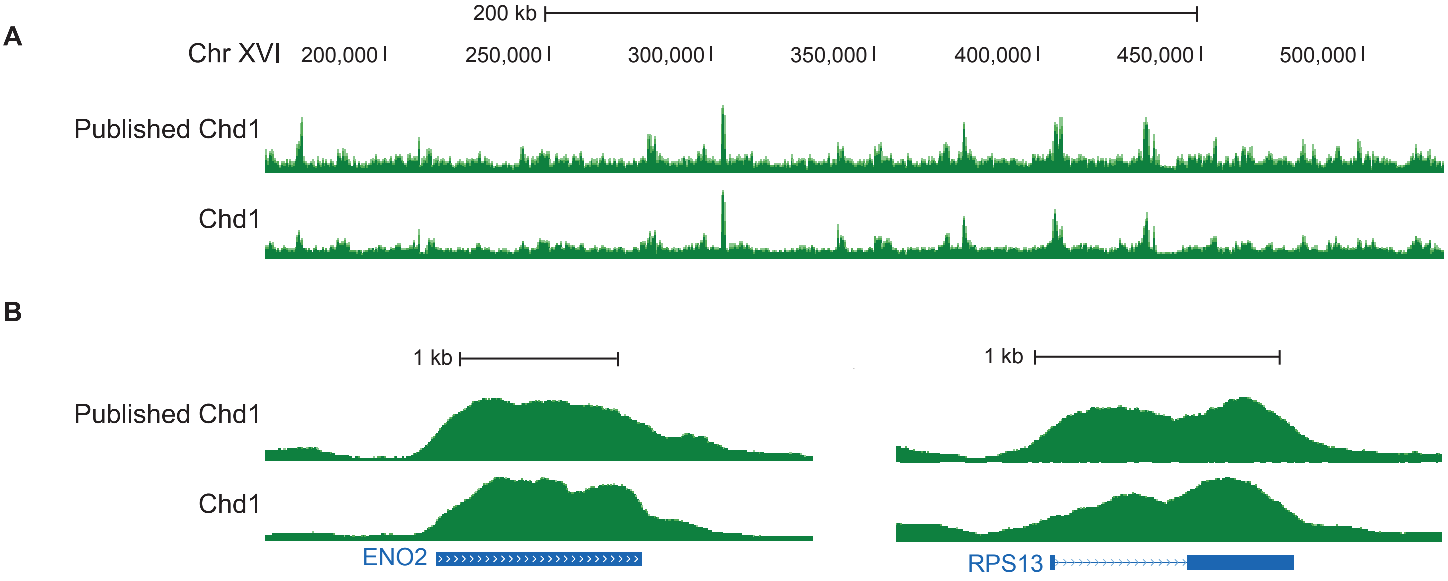
A
200 kb
Chr XVI
200,000
250,000
300,000
350,000
400,000
450,000
500,000
Published Chd1
Chd1
B
1 kb
Published Chd1
Chd1
ENO2
1 kb
RPS13

Figure S3

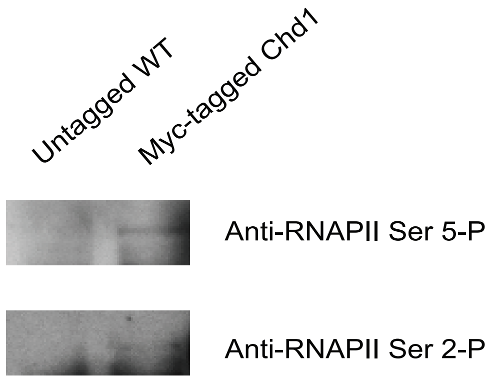

Untagged WT
Myc-tagged Chd1
Anti-RNAPII Ser 5-P
Anti-RNAPII Ser 2-P

Figure S4

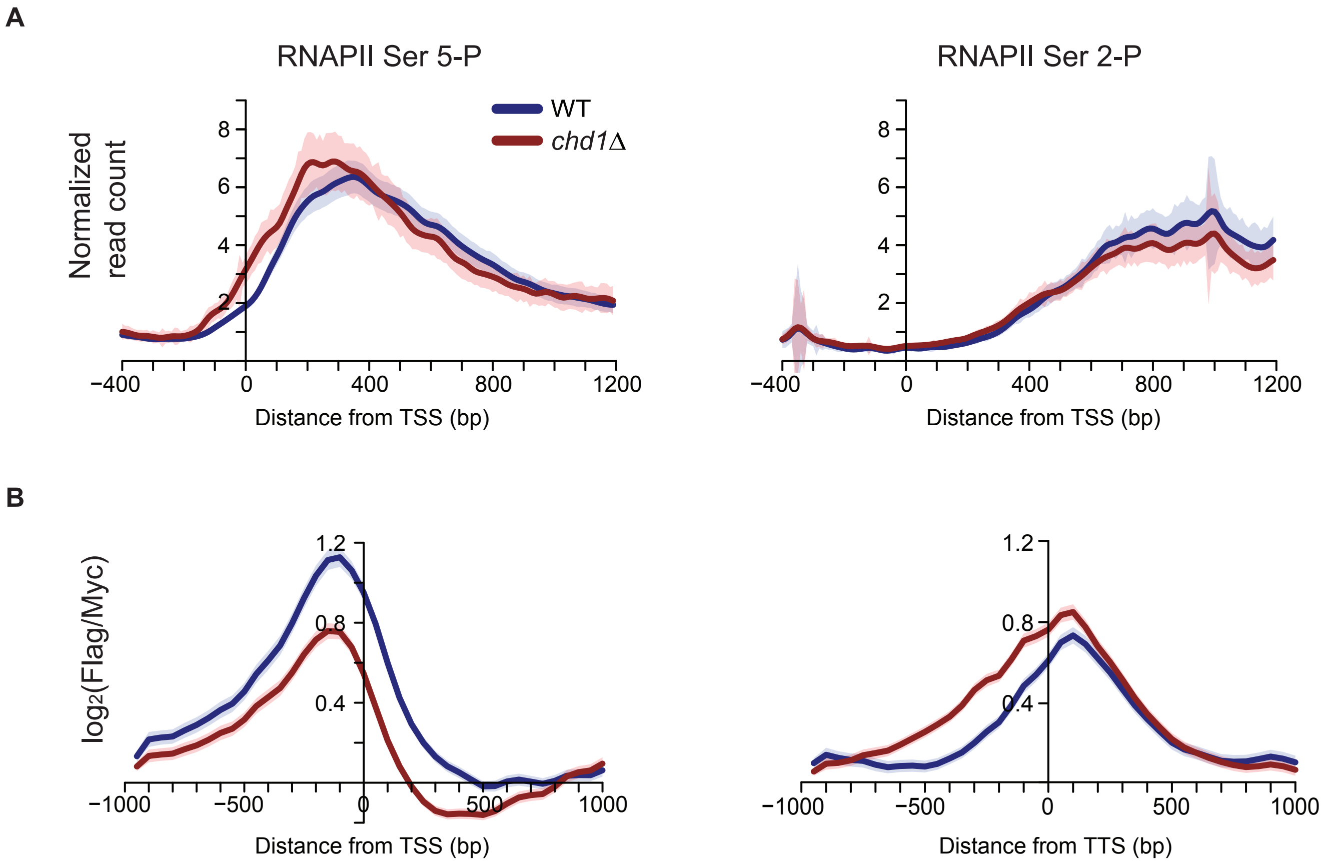
A
RNAPII Ser 5-P
RNAPII Ser 2-P
WT
chd1Δ
Normalized read count
8
6
4
2
−400
0
400
800
1200
Distance from TSS (bp)
B
log2(Flag/Myc)
1.2
0.8
0.4
−1000
−500
0
500
1000
Distance from TSS (bp)
Distance from TTS (bp)

Figure S5

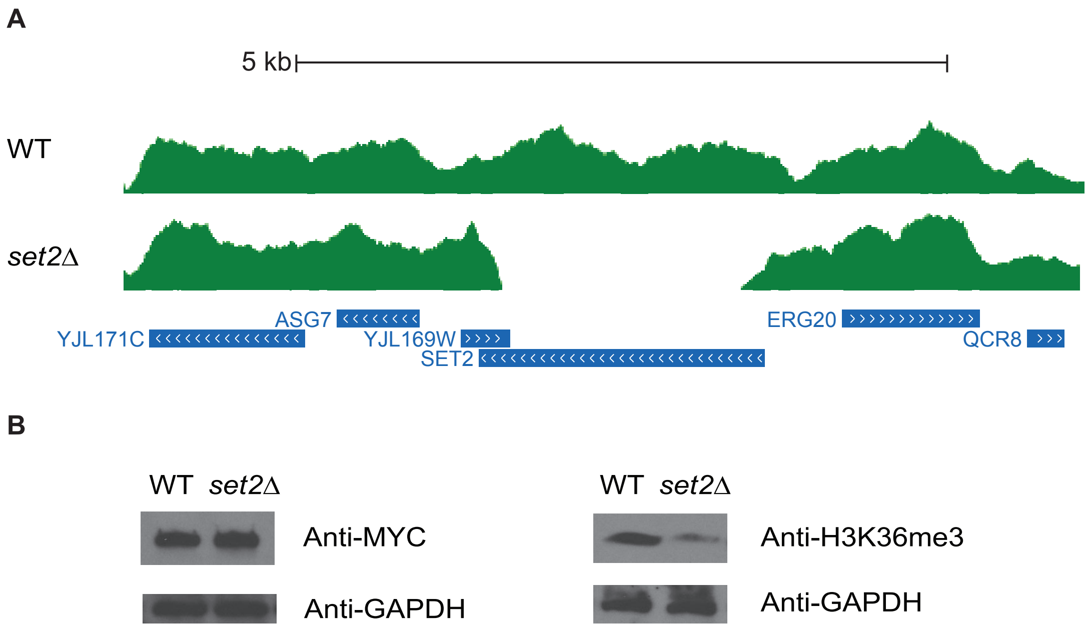
A
5 kb
WT
set2Δ
ASG7
YJL171C
YJL169W
SET2
ERG20
QCR8
B
WT set2Δ
Anti-MYC
Anti-GAPDH
WT set2Δ
Anti-H3K36me3
Anti-GAPDH

Figure S6

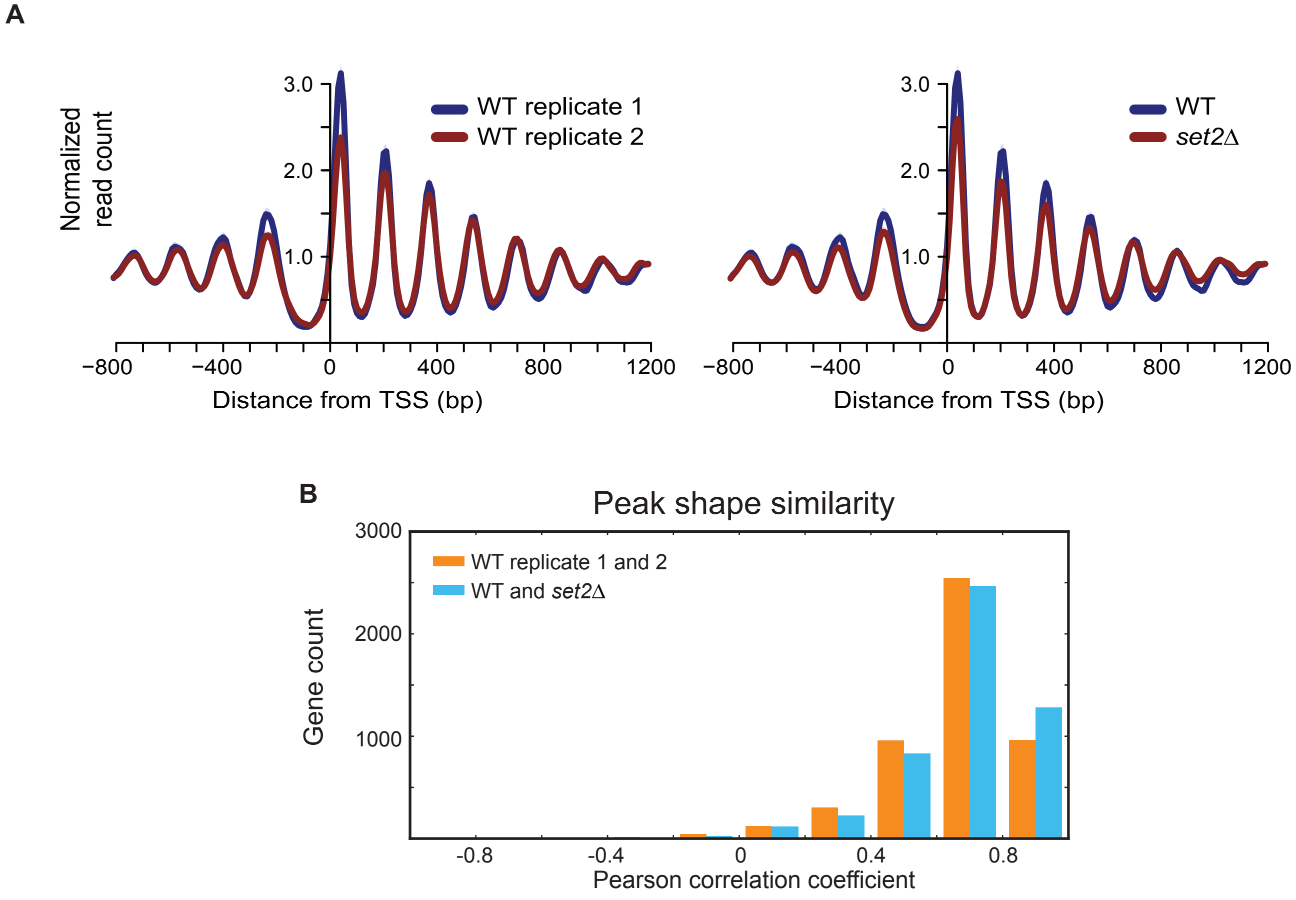
A
Normalized read count
3.0
2.0
1.0
WT replicate 1
WT replicate 2
−800
−400
0
400
800
1200
Distance from TSS (bp)
WT
set2Δ
B
Peak shape similarity
3000
2000
1000
WT replicate 1 and 2
WT and set2Δ
Gene count
-0.8
-0.4
0
0.4
0.8
Pearson correlation coefficient
